# Highlights of the FEBS3+ meeting *Exploring molecular frontiers*

**DOI:** 10.1002/2211-5463.70198

**Published:** 2026-02-04

**Authors:** Morana Dulić, Hannu Koistinen, Ronnie Berntsson

**Affiliations:** ^1^ Faculty of Science University of Zagreb Croatia; ^2^ Department of Clinical Chemistry and Haematology, Faculty of Medicine University of Helsinki and Helsinki University Hospital Finland; ^3^ Department of Medical Biochemistry and Biophysics Umeå University Umeå Sweden; ^4^ Wallenberg Centre for Molecular Medicine & Umeå Centre for Microbial Research Umeå University Umeå Sweden

## Abstract

FEBS+ meeting *Exploring molecular frontiers*, held in Pula, Croatia, in autumn 2024 was jointly organized by the Croatian, Finnish, and Swedish Member Societies of the Federation of European Biochemical Societies (FEBS). The congress covered a wide variety of different biochemical themes, some of the highlights of which are presented in this ‘In the Limelight’ issue of *FEBS Open Bio*. We hope that, in addition to being scientifically useful and interesting, these articles will give a glimpse of the excellent science presented in the FEBS3+ meeting *Exploring molecular frontiers* and encourage attendance to future FEBS3+ meetings.

**Abbreviations**
AI: Artificial Intelligence
EV: Extracellular vesicle
FEBS: Federation of European Biochemical Societies
PNPLA: Patatin‐like phospholipase
Trk: Tropomyosin receptor kinase

To increase international collaboration between molecular life scientists, the Federation of European Biochemical Societies (FEBS) supports scientific meetings, organized through collaboration of at least three FEBS Constituent Societies. Such FEBS3+ meetings are most often organized by neighboring countries. By the initiative of the Croatian Society for Biochemistry and Molecular Biology (HDBMB), which also acted as the main organizer, such a meeting was organized for the first time with geographically distant countries, that is, jointly with the Swedish Society for Biochemistry, Biophysics, and Molecular Biology (SFBBM) and the Finnish Biochemical, Biophysics, and Microbiology Society (Biobio Society). The FEBS3+ meeting, titled *Exploring molecular frontiers*, was organized on 25–28 September 2024 in Pula, Croatia (https://hdbmb.hr/en/congresses/), and attracted 185 participants from 17 countries. The scientific program covered a broad range of topics, from basic research to biomedicine and biotechnology. Life science education was also covered in an exciting session, offering insights into the use of rapidly developing technologies, such as Generative Artificial Intelligence (Gen AI) and virtual reality for teaching.

FEBS Junior Section (https://www.febs.org/events/early‐career‐meetings/febs‐junior‐section‐activities/) had a strong presence in the meeting. The purpose of the FEBS3+ meetings goes beyond just formal scientific sessions, so many possibilities for social interaction were tuned to promote collaborations and support the career development of young scientists. Hotel Park Plaza Histria served as an excellent venue to support such interactions throughout the day and during the social activities, like welcome reception and dinner.

To highlight the broad range of talks, this ‘In the Limelight’ issue of *FEBS Open Bio* includes seven contributions selected among four plenary lectures, 14 invited talks, 19 oral presentations, and 84 poster presentations. These contributions cover classical review articles offering novel insights and protocols that are foreseen to greatly benefit the bioscience field.

Among the review articles, André Mateus' and coworkers describe the progress in the structural and functional characterization of widespread protein O‐glycosylation in the Bacteroidota phylum [1]. This is an important field of research, not least because bacterial species in this phylum are a major component of the human gut microbiome, which has an important role in human health and disease. Despite the instrumental roles of protein glycosylation in multicellular organisms and widespread protein glycosylation in some bacterial phyla, the mechanisms and functions of glycosylation in bacteria are still rather poorly known as highlighted in the ‘*Future outlook*’ section of the review.

Giray Enkavi introduces structural and molecular mechanisms connecting ligand binding to the diverse signaling outcomes of tropomyosin receptor kinase (Trk) receptors [2]. These receptors interact with neurotrophins and are essential regulators of neuronal development, survival, and plasticity. As such, they are important drug targets. Since allosteric modulation of Trk receptor allows different neurotrophins binding to the same receptor to selectively activate specific downstream pathways, understanding of Trk receptor structure and dynamics is important for the development of therapeutic drugs.

Igor Weber *et al*. describe various properties of IqgC from *Dictyostelium discoideum*, supporting classification of this protein as a Ras GTPase‐activating protein (RasGAP) [3]. The group hypothesizes that IqgC regulates macroendocytosis and migration of amoeboid *D. discoideum* cells.

The patatin‐like phospholipase (PNPLA) domain‐vcontaining proteins are a group of enzymes with diverse activities and important roles, for example, in signaling pathways, membrane remodeling, lipid metabolism, and host colonization. Monika Oberer *et al*. offered a comprehensive review of the PNPLA proteins, their enzymatic activities, diversity, biological role, and regulatory mechanisms [4]. Understanding the interactions that regulate the activities of these enzymes can help in the elucidation of their roles in physiology and pathophysiology. Cellular RNAs fold into complex structures through base‐pairing and interact with proteins to form ribonucleoproteins, which play essential roles in gene regulation and are increasingly implicated in major human diseases. In this review, Minna‐Liisa Änkö and colleagues summarise methods for probing RNA–protein interactions and RNA structures [5]. They also illustrate how these approaches have deepened our understanding of ribonucleoproteins in gene regulation and discuss how such insights are opening new possibilities for drug development.

As noted in the Editorial by Ivana Novak for *FEBS Open Bio's* ‘In the Limelight’ issue, Research Protocols, ‘*FEBS Open Bio aims to highlight the critical importance of publishing reproducible and detailed scientific protocols that can be broadly adopted by laboratories working in molecular and cellular life sciences*’ [6]. In keeping with this aim, two research protocols have been selected in this issue.

Cardiovascular diseases are among the leading causes of morbidity and disability worldwide. While clot‐dissolving thrombolytic enzymes offer cost‐effective therapeutic intervention for these life‐threatening conditions, the effectiveness, and safety profiles of thrombolytic drugs need to be improved. To this end, Martin Toul *et al*. present protocols for the identification of thrombolytic enzymes and molecules affecting those [7]. These assays provide information on multiple relevant characteristics enabling fast and reliable evaluation of candidate effectiveness and other properties.

Extracellular vesicles (EVs) are lipid bilayer‐surrounded small vesicles released by almost all types of cells, including cancer cells. These carry and deliver to other cells bioactive molecules derived from the cells from which EVs originate. Since EVs are ubiquitously found in biofluids, they have gained a lot of interest, for example, as cancer biomarkers. However, the current methodology, such as ultracentrifugation, used for EV isolation is far from optimal. Caglar Elbuken and team provide protocols for electro‐viscoelastic microfluidics as a novel approach for manipulating EVs [8]. The system has potential for further development to be used for EV isolation.

We hope that, in addition to being scientifically useful and interesting, these articles will give a glimpse of the excellent science presented in the FEBS3+ meeting *Exploring molecular frontiers* and encourage attendance to the future FEBS3+ meetings (https://www.febs.org/febs‐societies/support‐for‐activities‐of‐febs‐societies/).

## Conflict of interest

The authors declare no conflict of interest.

## Author contributions

HK, RB, and MD wrote the editorial.
